# Genetic ancestry is associated with asthma, and this could be modified by environmental factors. A systematic review

**DOI:** 10.1111/cea.14308

**Published:** 2023-04-13

**Authors:** Jonathan Pham, Dinh S. Bui, Caroline J. Lodge, Michael J. Abramson, Adrian J. Lowe, Shuai Li, Aung K. Win, Mark Hew, Shyamali C. Dharmage

**Affiliations:** ^1^ Allergy and Lung Health Unit, Centre for Epidemiology and Biostatistics, School of Population & Global Health The University of Melbourne Melbourne Victoria Australia; ^2^ Asthma, Allergy and Clinical Immunology Service, Department of Respiratory Medicine Alfred Hospital Melbourne Victoria Australia; ^3^ School of Public Health & Preventive Medicine Monash University Melbourne Victoria Australia; ^4^ Centre for Epidemiology and Biostatistics, Melbourne School of Population and Global Health The University of Melbourne Parkville Victoria Australia; ^5^ Centre for Cancer Genetic Epidemiology, Department of Public Health and Primary Care University of Cambridge Cambridge UK; ^6^ Precision Medicine, School of Clinical Sciences at Monash Health Monash University Clayton Victoria Australia; ^7^ Centre for Molecular, Environmental, Genetic and Analytic Epidemiology, School of Population Health The University of Melbourne Melbourne Victoria Australia

**Keywords:** asthma, country, environment, ethnicity, genetic ancestry

**Key messages**

- While Native American genetic ancestry appears protective against asthma, African ancestry increases risk.
- There is indirect evidence that the latter is modified by self‐identified ethnicity, socio‐economics and country.
- Complex gene–environment interactions contribute to asthma in ethnic populations, highlighting the need for nuanced risk stratification.

To the editor,

Certain ethnic populations have a higher prevalence of asthma, a phenomenon which can be independent of socio‐economic status.^1^ Ethnicity is a product of genetic and cultural and behavioural factors. Using identified alleles in reference to genetic repositories, ‘genetic ancestry’ is a way of quantifying an individual's genetic composition inherited from ancestors of a particular geographic origin. *Kumar* et al. demonstrated that increased African genetic ancestry is independently associated with lower lung function.^2^ Genetic ancestry can lead to disease via epigenetic mechanisms. Evidence suggests DNA methylation can significantly differ between ethnic groups and this is induced by genetic ancestry and also environmental factors, such as tobacco smoke, air pollution and airborne pathogens.^3^

We systematically reviewed the evidence on associations between specific genetic ancestries and asthma, and environmental factors that could mediate or modify these associations. The study protocol was prospectively registered with PROSPERO (CRD42021222527). We searched MEDLINE, EMBASE and EBSCO Global Health for published studies that examined the association between genetic ancestry and asthma, to December 2022. We searched full‐text peer‐reviewed articles using a pre‐specified search strategy (exp asthma/OR asthma*.tw.) AND (exp ethnic groups/ OR ethnic*.tw. OR racial*tw. OR race.tw) AND (ancestr*.tw.). To be included, manuscripts defined asthma by physician diagnosis, ≥2 episodes of childhood wheeze, or using validated questionnaires, and quantified genetic ancestry on a continuous scale. We excluded manuscripts in which asthma severity or phenotypes were the outcome, rather than asthma risk. We included manuscripts that estimated the proportion of genetic ancestry using admixture mapping of participants' genome‐wide data. Genetic ancestry can be determined by quantifying the number of previously identified alleles in reference populations that strongly correlate with ancestral origins of a specific geographic origin (e.g., Europe, Africa, etc.)—known as *ancestry informative markers* (AIMs). Analysis of an individual's genetic data allows quantification of AIMs and, therefore, estimation of proportions of genetic ancestry.

Screening, data extraction and assessment of bias were conducted independently by two reviewers, (JP and DB) with a third reviewer adjudicating any discrepancies (SD). Where a manuscript included multiple cohorts, results were reported for individual cohorts, if available, otherwise, the reported aggregated effect estimates were used. Bias was assessed using the Newcastle‐Ottawa Scale, with studies scoring less than 5 deemed at significant risk of bias. Due to substantial clinical and methodological heterogeneity across studies, meta‐analysis was not performed. Additional information about study methods and findings is available in the following online repository https://doi.org/10.5281/zenodo.7693977.

From 114 records, 9 manuscripts (14 independent cohorts) fulfilled the eligibility criteria. Most eligible manuscripts (7 of 9) investigated African ancestry. Other ancestries were European (*n* = 6), Native American (*n* = 3), Asian (*n* = 1) and Iberian (*n* = 1). Most manuscripts (6 of 9) investigated individuals residing in the United States; the remaining three investigated individuals living in Brazil, Canary Islands and Peru. No studies were assessed as having a significant risk of bias.

As shown in Figure 1, Native American ancestry was associated with a lower risk of asthma in three manuscripts (Hispanic Americans: two different cohorts: OR 0.92 and 0.85, per 10% increase in Native American ancestry; one cohort *p* < .05. Peruvians: one cohort: OR 0.87, *p* = .12). In self‐identified African‐American populations, every 10% increase in African ancestry was associated with an increased risk of asthma in two manuscripts with three different cohorts: (OR 1.10–10.1; two reached significance at *p* < .05). In contrast, among self‐identified Hispanic populations living in the USA, African ancestry was associated with nil‐to‐modestly increased asthma risk in two manuscripts with three different cohorts: (OR 1.18 and 1.18, one reached significance at *p* < .05). The manuscript by *Choudhry* et al. also investigated African ancestry among Hispanic Americans, but the result could not be compared with others, as it did not report relevant estimates. Nonetheless, this manuscript did not show any significant association between African ancestry and asthma. There was no association between African ancestry and asthma in Caribbean countries in one manuscript with two different cohorts (Jamaica and Barbados): OR 0.80–1.10, respectively, compared to countries in South America that showed significant associations (Brazil: two manuscripts, two different cohorts: OR 1.11 and 137; *p* < .05. Colombia: one manuscript: OR 4.50, *p* < .05).

**FIGURE 1:**
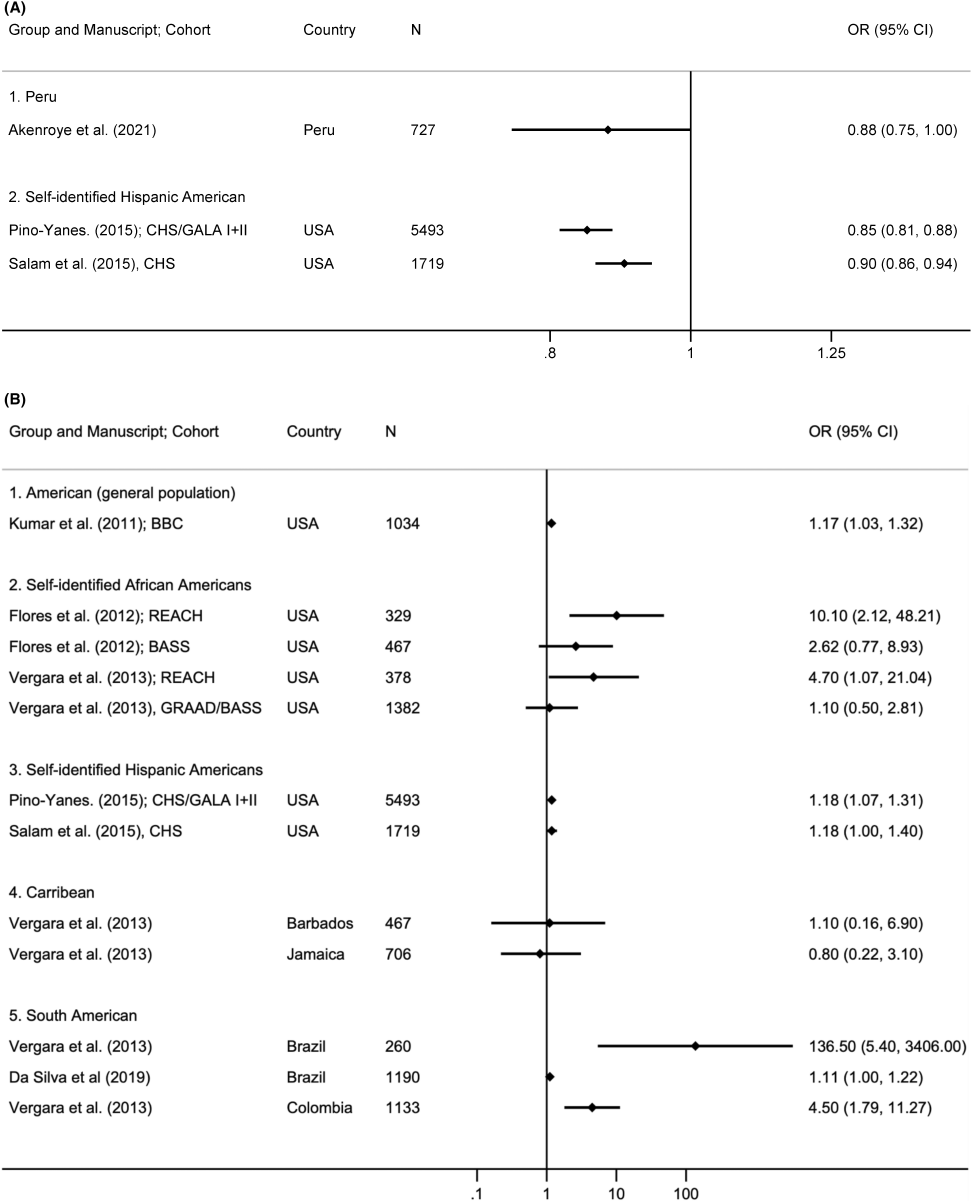
(A) Asthma risk per 10% increase in Native American ancestry, among different populations. (B) Asthma risk per 10% increase in African ancestry, among different populations.

Two of three manuscripts examining European ancestry in Americans reported no association with asthma (a self‐identified Hispanic American cohort: OR 1.06, *p* > .05; a self‐identified African American cohort: no OR or p value reported). One manuscript showed only a modest inverse association between European ancestry and asthma (multi‐ethnic cohort: OR 0.84; *p* < .05). A single manuscript reported no association between Asian ancestry and asthma among Hispanic Americans (OR 1.06, *p* > .05). A single manuscript did not show an association between Iberian ancestry (compared to North African ancestry) and asthma in the Canary Islands (OR not reported, *p* > .05).

Two manuscripts in our review formally investigated potential effect modifiers. *Choudhry* et al. found that among Puerto Ricans, the association between African ancestry and asthma was only observed in individuals from higher, but not in lower socio‐economic strata (interaction *p* = .005).

*Salam* et al. investigated acculturation as a potential effect modifier, using the mother's birthplace and parental language preference as proxies, however, this did not influence the association between Native American ancestry and asthma in self‐identified Hispanic Americans. African ancestry was associated with asthma in South America but not in Caribbean countries.

This is the first systematic review to synthesize evidence on asthma risk for different genetic ancestries. The current body of evidence highlights positive and negative associations between genetic ancestry and asthma and identifies possible effect modifiers for this relationship. Native American genetic ancestry was protective from asthma, whereas other ancestries were not. One explanation is that certain genetic ancestries have better immunological adaptation to specific environments. Migration could also play a role, either in association or independently. A study of immigrants to Australia suggested that such individuals were more likely to develop a non‐eosinophilic asthma phenotype compared to Australian‐born individuals, suggesting that migrating to new environments may induce certain immunological processes.^4^

The association between asthma and African genetic ancestry was amplified among African Americans and in South America but attenuated among Hispanic Americans and in Caribbean countries, suggesting effect modification or mediation by environmental factors. Interactions between African genetic ancestry and environmental factors have also been demonstrated in other non‐communicable diseases. In a genetic study of Afro‐Colombian populations, African genetic ancestry correlated well with validated polygenetic risk scores for type 2 diabetes^5^ but that genetic ancestry alone was insufficient to explain the different prevalence of diabetes across various Colombian localities, suggesting that environmental factors such as diet and socio‐economics are also important.

Genetic ancestry was associated with asthma in high, rather than low, socio‐economic strata. One possible explanation is that high socio‐economic status may lead to increased hygiene and reduced levels of protective microbiologic exposure in early life. This has been described among the Hutterites with decreased childhood exposure to airborne animal endotoxins and higher levels of asthma compared to the genetically similar Amish population.^6^

Our systematic review has identified certain populations vulnerable to asthma, owing to a combination of genetic and socio‐demographic factors, likely to induce gene–environment interactions between genetic ancestry, country of residence, cultural affiliation and socio‐economic status. These findings could help inform future case findings and public health interventions by identifying high‐risk socio‐demographic profiles, supported by genetic ancestry data. In addition, further genetic analysis within such high‐risk profiles could yield future genetic targets for personalized medicine. For instance, recent data among Hispanic Americans demonstrate that Native American genetic ancestry at chromosomal region 18q21 (upstream of the SMAD2 gene) is associated with excess asthma risk.^7^ Given that SMAD2 has also been implicated in transforming growth factor beta (TGFβ) signal transduction in asthma,^8^ it is possible that Hispanic Americans bearing this genotype may respond to therapies targeting TGFβ.

One major limitation of this review is the paucity of genetic cohorts available, small sample sizes and heterogeneity of findings. Rapid advances in genome‐wide genotyping methods have improved the understanding of complex trait illnesses such as asthma. However, the majority of genetic cohort studies are from European ancestry populations, representing a significant knowledge gap.^9^ Empirically applying disease‐predictive algorithms to ethnic populations is not only inappropriate but has also been shown to give rise to spurious results.^9^ Heterogeneity of effect estimates in our review could be explained by differences in sampled sub‐ethnic subpopulations and/or genetic reference measurements. For instance, Hispanic American subpopulations differed between studies, such as Puerto Rican versus Mexican American, who could exhibit different environmental exposures. Furthermore, genetic ancestry measurement was not standardized across studies which could impact comparability. For example, African genetic ancestry was drawn from different populations, such as the Yoruba in West Africa (Nigeria) and Luhya in East Africa (Kenya).

In summary, genetic ancestry is associated with asthma and this could be modified by cultural affiliation, country of residence and socio‐economics. Our findings identify socio‐demographic profiles which, supported by genetic ancestry data, could be targeted for additional public health intervention and case finding. Further research to identify genetic targets for therapeutic intervention, catering for at‐risk genetic profiles, may alleviate asthma inequality across ethnic populations.

## CONFLICT OF INTEREST STATEMENT

I confirm that no conflict of interest exists for the specified authors.

## PROSPERO REGISTRATION

CRD42021222527 at www.crd.york.ac.uk/prospero/.

## SYSTEMATIC REVIEW REGISTRATION

An open‐access search strategy and study protocol were registered with PROSPERO (CRD42021222527).

## CONFERENCE PRESENTATION

American Thoracic Society 2021 international conference (virtual), poster presentation, 14 May 2021.

## Data Availability

The data that support the findings of this study are available from the corresponding author upon reasonable request.
